# Acute Intermittent Porphyria: A Rare Cause of Postoperative Abdominal Pain and Hyponatremia

**DOI:** 10.7759/cureus.91823

**Published:** 2025-09-08

**Authors:** Shafayath Chowdhury, Brandon Weissman, Constantino G Lambroussis

**Affiliations:** 1 Anesthesiology and Critical Care, Lake Erie College of Osteopathic Medicine, Elmira, USA; 2 Otolaryngology, Lake Erie College of Osteopathic Medicine, Elmira, USA; 3 Osteopathic Medicine/Family Medicine, Lake Erie College of Osteopathic Medicine, Elmira, USA

**Keywords:** acute hepatic porphyria, acute intermittent porphyria (aip), anesthesia complication, general anesthesia, laparoscopic cholecystectomy

## Abstract

Acute hepatic porphyrias (AHP) are a group of rare metabolic disorders that develop from heme biosynthesis enzyme deficiencies. They often result in the accumulation of neurotoxic intermediates, such as aminolevulinic acid (ALA) and porphobilinogen (PBG). The disorders can be triggered by specific medications, prolonged fasting, or physiological stress. We present a case of a 34-year-old female patient with no significant medical conditions or comorbidities, who started experiencing severe abdominal pain, tachycardia, and hyponatremia after undergoing laparoscopic cholecystectomy under general anesthesia. The diagnosis of acute intermittent porphyria (AIP), a subtype of AHP, became evident with elevated urinary ALA and PBG upon laboratory testing. The patient received intravenous hemin treatment along with supportive care, which resulted in rapid resolution of symptoms. The case demonstrates why healthcare providers should include porphyria in their diagnostic considerations when patients present with unexplained postoperative abdominal pain and hyponatremia, especially when anesthetic triggers are involved. Early diagnosis through targeted therapy halted neurological deterioration and reduced the duration of hospital stay. Heightened awareness among anesthesiologists and perioperative clinicians would lead to earlier detection and improved outcomes for patients with similar conditions.

## Introduction

Acute hepatic porphyrias (AHP) represent rare inherited metabolic disorders that occur in one in 100,000 people [1]. These occur when the heme biosynthesis pathway contains deficient enzymes that lead to toxic intermediate accumulation. The most prevalent form of AHP is acute intermittent porphyria (AIP), which is caused by porphobilinogen deaminase enzyme deficiency [2]. The clinical symptoms of AHP vary widely, but patients commonly experience severe abdominal pain combined with autonomic instability, neuropsychiatric symptoms, and hyponatremia caused by the syndrome of inappropriate antidiuretic hormone secretion (SIADH) [3].

The onset of an AHP attack occurs when patients experience stress from fasting, hormonal changes, infections, or take specific medications that can serve as triggers. Medications that could potentially trigger an AHP attack include barbiturates, sulfonamides, and certain anesthetic agents such as etomidate and ketamine [4]. There is often a delay in diagnosis of these presentations because they can mimic common surgical or postoperative complications. This delay in diagnosis can result in severe neurological damage or fatal outcomes due to the rapidly progressive nature of untreated attacks [5]. The anesthesiologist plays an essential role here because fasting periods, surgical stress, and medication administration during the perioperative period can serve as triggering factors for an attack. Knowledge about safe versus unsafe anesthetic options in porphyria patients would decrease the risk of operative complications. We present the case of a female patient with postoperative abdominal pain, hyponatremia, and lab findings that were diagnostic for an acute AIP attack.

## Case presentation

A 34-year-old woman with no known medical conditions presented to the hospital for an elective laparoscopic cholecystectomy due to symptomatic cholelithiasis. The preoperative evaluation was unremarkable with a basic metabolic panel, complete blood count, and liver function tests within normal limits. General anesthesia was induced with propofol, fentanyl, and rocuronium, followed by maintenance with sevoflurane. The surgery itself was uneventful and lasted a total duration of 90 minutes.

The patient developed severe diffuse abdominal pain out of proportion to exam findings, tachycardia, mild hypertension, and agitation approximately 12 hours postoperatively. She had no fever, leukocytosis, or peritoneal signs. A surgical complication was initially suspected; however, laboratory evaluation revealed hyponatremia and mild transaminase elevation. Serial hemoglobin and white blood cell counts remained stable, and repeat clinical examinations did not demonstrate any signs of peritonitis. No acute changes were observed on postoperative abdominal imaging, which further lowered suspicion for a surgical etiology. Urine output was monitored, and no discoloration or diuresis was observed. Fluid balance was also documented, which revealed urine and plasma osmolality to be consistent with SIADH as the likely cause of hyponatremia.

The patient stated that she had experienced similar episodes of abdominal pain in the past. These episodes occurred intermittently during the previous year and resolved spontaneously without medical intervention. No obvious trigger had been identified during those prior episodes. There was no family history of porphyria, unexplained early death, or similar postoperative complications. Given the unexplained symptoms and hyponatremia, an AHP was suspected. Subsequently, urine porphobilinogen (PBG) and aminolevulinic acid (ALA) were measured and found to be markedly elevated, confirming the diagnosis of acute intermittent porphyria. Relevant laboratory findings on postoperative day one are displayed in Table 1.

**Table 1:** Laboratory values on postoperative day one K/uL: thousands per microliter, g/dL: grams per deciliter, fL: femtoliters, mg/dL: milligrams per deciliter, mmol/L: millimoles per liter, U/L: units per liter, mOsm/kg: milliosmoles per kilogram, mg/L: milligrams per liter, AST: aspartate aminotransferase, ALT: alanine aminotransferase, PBG: porphobilinogen, ALA: aminolevulinic acid

Parameter	Result	Reference Range
White blood count	9.5 K/uL	4.0-10.5 K/uL
Red blood count	4.8 K/uL	4.70-6.0 K/uL
Hemoglobin	15.2 g/dL	14.0-18.0 g/dL
Hematocrit	45.2%	42-52%
Mean corpuscular volume	90.1 fL	78.0-100.0 fL
Glucose level	95 mg/dL	60.0-100.0 mg/dL
BUN	18 mg/dL	7-20 mg/dL
Creatinine	1.21 mg/dL	0.4-1.40 mg/dL
Sodium level	122 mmol/L	135-140 mmol/L
Potassium level	3.9 mmol/L	3.5-5.0 mmol/L
AST	58 U/L	0-35 U/L
ALT	66 U/L	0-45 U/L
Plasma osmolality	260 mOsm/kg	275-295 mOsm/kg
Urine osmolality	420 mOsm/kg	50-1200 mOsm/kg
Urine sodium	55 mmol/L	20-40 mmol/L
Urine PBG	45 mg/L	<2 mg/L
Urine ALA	36 mg/L	<7 mg/L

The patient was started on intravenous hemin for four days, as well as intravenous normal saline for correction of hyponatremia and opioids for pain management. The patient received instructions to stay away from medications known to trigger porphyria. The patient received significant symptom improvement during the first 48 hours of treatment and was discharged home on postoperative day six with outpatient genetic counseling.

## Discussion

Our case presentation demonstrates the difficulties of diagnosing AHP in a perioperative environment, especially in the context of an absence of any comorbidities in our patient. Our patient presented with severe abdominal pain, tachycardia, and hyponatremia, with no clear signs suspicious of surgical complications or electrolyte disorders from other sources. The neuropathic abdominal pain in AHP develops from neurotoxic heme precursors that affect the autonomic nervous system [5].

The perioperative period poses a high risk for porphyria attacks because fasting, surgical stress, and anesthetic agents can trigger these attacks [4,6]. The patient experienced a porphyria episode because of the combined effects of surgical stress and general anesthesia. Although propofol and sevoflurane are generally considered safe for porphyria patients, careful perioperative management is essential. This is because even anesthetics deemed to be “safe” and physiological stress can still precipitate acute attacks if not properly managed [7].

The condition of hyponatremia in porphyria patients occurs due to SIADH and affects 40% of patients during attacks [2]. If the condition remains untreated, it may cause patients to develop sequelae of hyponatremia, such as seizures or a coma. The primary treatment for acute attacks involves intravenous hemin administration because it reduces hepatic aminolevulinic acid synthase (ALA-S) activity. This enzyme controls heme synthesis, and inhibition of the enzyme would decrease toxic precursor accumulation [6,8].

The identification of porphyria requires immediate attention because delayed medical intervention may lead to permanent neurologic damage. Our case showcases that postoperative abdominal pain combined with hyponatremia should prompt healthcare providers to consider porphyria in their differential diagnosis. It also highlights the necessity to stray away from cognitive biases that may have delayed the diagnosis in this case because the patient presented with an insignificant past medical history. This case illustrates the diagnostic challenge of acute intermittent porphyria in a young, otherwise healthy patient undergoing a routine laparoscopic cholecystectomy. The absence of comorbidities or obvious anesthetic risk factors can easily lower clinical suspicion, highlighting the need for a high index of suspicion in similar presentations.

## Conclusions

Acute hepatic porphyria represents a rare but severe surgical complication, which presents with symptoms that resemble typical postoperative complications. The diagnosis requires strong clinical suspicion, especially when hyponatremia appears without identifiable causes. The ability of anesthesiologists to prevent and detect attacks becomes crucial since any small stressor during surgery or medication can trigger these attacks. The combination of urinary PBG and ALA testing for diagnosis, followed by prompt hemin therapy, becomes essential for saving patients' lives. The case demonstrates why healthcare providers must conduct detailed preoperative interviews because even the most minor of prior symptoms could be an indication for underlying porphyria. The prevention of future attacks and long-term prognosis improvement depends on ongoing patient education and the avoidance of known triggers.
